# Limited but powerful toolbox: myeloid cells as critical immunomodulators in glioma progression

**DOI:** 10.1038/s41392-025-02275-y

**Published:** 2025-06-11

**Authors:** Yun Liu, Jincheng Fang, Roman Sankowski

**Affiliations:** Institute of Neuropathology, Faculty of Medicine, University of Freiburg, Freiburg, Germany

**Keywords:** Tumour immunology, CNS cancer

In their recent publication in Nature, Miller et al. studied recurrent transcriptional programs across various brain tumors. The authors identified four immunomodulatory expression programs in myeloid cells driven by factors within the tumor microenvironment (TME) including hypoxia, IL-1β, TGFβ, and dexamethasone treatment^1^. Notably, the authors link clinical use of dexamethasone with an immunosuppressive myeloid program, potentially highlighting dexamethason’s compounding effect on glioblastoma-associated immunosuppression.

Dexamethasone is currently a mainstay therapy used to control peritumoral edema. The identified myeloid programs can—to some extent—predict immunotherapy response and survival rates, providing new insights for the regulatory myeloid cell landscape. Among many innovations, the central groundbreaking aspect of the study is that the tumor microenvironment is the main driver of myeloid states. Earlier evidence could not exclude the role of cell ontogeny (brain-resident vs. bone marrow-derived) and tumor characteristics (e.g., certain mutations). The findings highlight avenues towards developing novel immunotherapies that should focus on shielding immune cells from the tumor microenvironment.

**Fig. 1:**
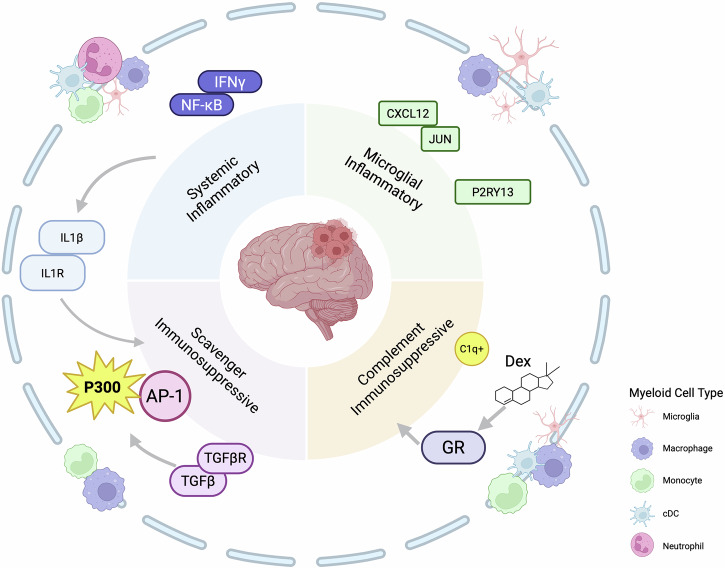
Schematic diagram of the four immunomodulatory activity programs in glioma-associated myeloid cells, along with the associated microenvironmental correlations and perturbation factors. IL-1β and TGFβ induced the scavenger immunosuppressive program. Dexamethasone and IFNγ induced the complement immunosuppressive program and the systemic inflammatory program, respectively. The p300 inhibitor downregulates the AP-1-dependent scavenger immunosuppression. The figure was created with BioRender.com

Glioblastomas are the most prevalent and lethal malignant brain tumor, mainly due to a unique immune TME^2^. Myeloid cells comprising about half of the total tumor cell content, create a highly immunosuppressive environment that hinders an effective immune response. While malignant cell states in glioblastoma have been extensively mapped^3^, the identity and regulatory programs of myeloid populations remain an area of active research. The complexity of brain-resident macrophage populations and blood-derived engrafting cells makes decoding the states and origins of glioblastoma-associated macrophages particularly challenging^4^. Various interactions between myeloid, tumor and stromal cells add further layers of complexity. To investigate these issues, Miller et al. examined published single-cell RNA sequencing data from 85 human glioma specimens, using a consensus non-negative matrix factorization (cNMF). The authors selected this method for its robustness in identifying transcriptional programs compared to commonly used similarity-based clustering approaches. They expanded these analyses with mitochondrial lineage tracing, spatial transcriptomics, chromatin accessibility analysis, and experimental glioblastoma organoid models. Comparative analyses across various tumor entities—including IDH-mutant, primary and recurrent gliomas—and different treatment conditions demonstrated that the identified programs persist across these variables. Specifically, they discover four key immunomodulatory programs: systemic inflammatory, microglial inflammatory, Scavenger immunosuppression, and complement immunosuppression (Fig. 1). These programs are consistently present across myeloid cell populations while exhibiting distinct usage patterns depending on the ontogeny.

Underscoring the role of myeloid cells, the study revealed that immunomodulation-related gene expression patterns were the most common functional patterns in glioma-associated myeloid cells, with 91% involved in at least one of the identified immunomodulatory programs. For example, blood-derived macrophages can express all four immunomodulatory programs but are mainly enriched in two immunosuppressive programs, while brain-resident microglia rarely express scavenger immunosuppressive programs. Moreover, this difference is also observed between CNS and non-CNS tumors, exemplified by carcinoma metastases. Systemic inflammatory programs and complement immunosuppressive programs are present in gliomas and metastases, whereas microglial inflammatory programs and scavenger-related immunosuppressive programs are primarily specific to gliomas, with minimal expression in brain metastases. Additionally, the researchers found that all immunomodulatory programs except the scavenger immunosuppressive program were detectable in non-neoplastic brain tissue, further suggesting that the scavenger program may be specific to primary CNS tumors. These findings underscore the immunosuppressive features of glioma-associated macrophages.

To further characterize the spatial distribution of myeloid cells within the TME, the researchers mapped their single-cell data onto published Visium spatial transcriptomic data^2^. The authors identified six major TME regional programs, with different myeloid gene programs exhibiting distinct spatial distributions: scavenger immunosuppressive programs were enriched in vascular and hypoxic regions, while complement immunosuppressive programs showed lower hypoxic expression but predominate in inflammatory and vascular regions. Systemic inflammatory programs are primarily localized in hypoxic and inflammatory areas, whereas microglial inflammatory programs concentrate in inflammatory and vascular zones.

To further validate these findings, the authors co-cultured glioma organoids (GBO) with matched peripheral blood mononuclear cells. After 1 week, GBOs exhibited extensive myeloid cell infiltration. Notably, a subset of infiltrated myeloid cells displayed mixed macrophage-like and microglia-like characteristics, whereas non-infiltrated myeloid cells expressed significantly lower levels of microglia-associated genes, including *TMEM119* and *P2RY12*. These results confirmed prior work showing that blood-derived monocytes can adopt microglia-like phenotypes upon entering the TME. This contrasts with monocytes, which exclusively expressed systemic inflammatory programs. These findings further demonstrate myeloid plasticity with their immunomodulatory state strongly influenced by the TME. This adaptation is critical for the immune regulation in tumors.

Next, the authors pinpointed the transcriptional programs involved in the clinical response to immunotherapy. Patients with a higher activity of the scavenger immunosuppressive program exhibited poor response to PD-1 blockade therapy. Consistently, elevated expression of the scavenger immunosuppressive program was significantly correlated with reduced overall survival. The AP-1 signaling pathway proved critical for scavenger immunosuppressive programs and could be selectively suppressed by inhibiting the p300/CBP pathway with the compound GNE-781, while simultaneously augmenting systemic inflammatory responses. This finding highlights a potential approach to reprogramming myeloid cells toward a more immune-activating phenotype, potentially enhancing anti-tumor immunity. Another key finding was that while dexamethasone alleviates brain edema in glioma patients, it also exacerbates durable myeloid-driven immunosuppression. Given the persistence of this effect, the study suggests that corticosteroid use should be approached with caution.

Although this study provides numerous novel insights into myeloid cells and glioma, certain limitations remain. The analyses presented in this paper are primarily based on intraoperative tumor resections, and longitudinal data on the temporal dynamics of myeloid cell states in the same patient would be desirable^5^. Future studies could utilize time-series single-cell sequencing or in vivo lineage-tracing models to track the dynamic transformation of myeloid cells during tumor progression. Furthermore, this study primarily relies on organoid models to recapitulate aspects of the glioma microenvironment but does not fully capture the complexity of tumor-immune interactions in vivo. Additionally, while the study found p300/CBP inhibition to be a promising therapeutic strategy, its efficacy remains to be validated in preclinical in vivo models. Future studies should focus on mechanistic evaluation of the impact of myeloid-targeted therapy in animal models and patient cohorts, while exploring potential synergies with existing immunotherapies.

In conclusion, this study highlights the crucial impact of the tumor microenvironment on suppressing anti-tumor properties of glioma-associated myeloid cells. Future therapies will need to focus on biomarker-based patient stratification and myeloid-targeted immunotherapies. Most critically, these therapies can target myeloid cells while in circulation to prepare them for the harsh glioma microenvironment they are about to encounter. By defining four actionable myeloid states, the authors break down the biological complexity of what was formerly known as glioblastoma “multiforme”.
